# Effects of enzyme inducers efavirenz and tipranavir/ritonavir on the pharmacokinetics of the HIV integrase inhibitor dolutegravir

**DOI:** 10.1007/s00228-014-1732-8

**Published:** 2014-08-23

**Authors:** Ivy Song, Julie Borland, Shuguang Chen, Phyllis Guta, Yu Lou, David Wilfret, Toshihiro Wajima, Paul Savina, Amanda Peppercorn, Stephen Castellino, David Wagner, Louise Hosking, Michael Mosteller, Justin P. Rubio, Stephen C. Piscitelli

**Affiliations:** 1 GlaxoSmithKline, 5 Moore Drive, Research Triangle Park, Durham, NC 27709 USA; 2 GlaxoSmithKline, Stockley Park, Uxbridge, UK; 3 GlaxoSmithKline, Stevenage, UK; 4 Shionogi & Co., Ltd., Osaka, Japan

**Keywords:** Dolutegravir, Drug interaction, Efavirenz, Tipranavir

## Abstract

**Purpose:**

Dolutegravir (DTG) is an unboosted, integrase inhibitor for the treatment of HIV infection. Two studies evaluated the effects of efavirenz (EFV) and tipranavir/ritonavir (TPV/r) on DTG pharmacokinetics (PK) in healthy subjects.

**Methods:**

The first study was an open-label crossover where 12 subjects received DTG 50 mg every 24 hours (q24h) for 5 days, followed by DTG 50 mg and EFV 600 mg q24h for 14 days. The second study was an open-label crossover where 18 subjects received DTG 50 mg q24h for 5 days followed by TPV/r 500/200 mg every 12 hours (q12h) for 7 days and then DTG 50 mg q24h and TPV/r 500/200 mg q12h for a further 5 days. Safety assessments and serial PK samples were collected. Non-compartmental PK analysis and geometric mean ratios and 90 % confidence intervals were generated.

**Results:**

The combination of DTG with EFV or TPV/r was generally well tolerated. Four subjects discontinued the TPV/r study due to increases in alanine aminotransferase that were considered related to TPV/r. Co-administration with EFV resulted in decreases of 57, 39 and 75 % in DTG AUC_(0–*τ*)_, *C*_max_ and *C*_*τ*_, respectively. Co-administration with TPV/r resulted in decreases of 59, 46 and 76 % in DTG AUC_(0–*τ*)_, *C*_max_ and *C*_*τ*_, respectively.

**Conclusions:**

Given the reductions in exposure and PK/pharmacodynamic relationships in phase II/III trials, DTG should be given at an increased dose of 50 mg twice daily when co-administered with EFV or TPV/r, and alternative regimens without inducers should be considered in integrase inhibitor-resistant patients.

**Electronic supplementary material:**

The online version of this article (doi:10.1007/s00228-014-1732-8) contains supplementary material, which is available to authorized users.

## Introduction

Dolutegravir (DTG) is a potent, low-nanomolar inhibitor of HIV integrase. Studies in healthy subjects demonstrate that DTG is well tolerated, has low to moderate pharmacokinetic (PK) variability and achieves therapeutic concentrations with once-daily dosing [1]. Phase III studies in various patient populations demonstrate that DTG has a desirable safety/tolerability profile and sustained antiviral activity in combination with other antiretroviral agents in integrase inhibitor (INI)-naive- as well as INI-resistant, HIV-infected adults [2–5].

Dolutegravir is metabolised primarily through UDP-glucuronosyltransferase (UGT) 1A1 with a minor component (∼10 %) via CYP3A4 [6]. No clinically significant effects on DTG exposure requiring a dose adjustment have been observed with UGT1A1 and CYP3A4 inhibitors [7, 8]. However, a large decrease in exposure was observed when DTG was co-administered with the CYP3A4 inducer, etravirine [9]. Therefore, it was necessary to evaluate potential interactions with other antiretroviral agents possessing enzyme-inducing properties. Efavirenz (EFV) is a commonly used drug in HIV-infected individuals. Tipranavir combined with ritonavir (TPV/r) is less commonly used and generally limited to HIV-infected individuals with limited treatment options due to drug resistance. As both drugs have potential to induce drug-metabolising enzymes, and because DTG is being studied across a wide range of HIV-infected populations, drug interaction studies with both medications were warranted.

In vitro, DTG demonstrates minimal or no direct inhibition of CYP isozymes, UGT1A1, UGT2B7 and many transporters [P-glycoprotein (P-gp), BCRP, OATP1B1, OATP1B3, MRP2], and is not an inducer of CYP1A2, CYP2B6 or CYP3A4 [10]. Dolutegravir also had no significant effect on midazolam exposure in healthy subjects [1]. Therefore, the primary objective was to evaluate the effect of EFV and TPV/r on DTG PK in healthy subjects, not vice versa.

## Methods

Two studies were conducted in healthy volunteer subjects as judged by physical exam, medical history and laboratory testing. These studies are registered with ClinicalTrials.gov (NCT01068925; protocol number ING113096 and NCT01098526; protocol number ING114005). Adult men and women of non-childbearing potential were enrolled. Subjects were excluded if they tested positive for HIV or hepatitis C antibodies or hepatitis B surface antigen. Subjects were not allowed to receive any prescription or non-prescription drugs, including vitamins or herbal products, within 7 days of dosing and throughout the study. Subjects had a screening visit (within 30 days prior to the first dose of study drug), treatment periods and a follow-up visit (7–14 days after the last dose of study drug).

### Interaction with EFV

This was a phase I, open-label, single-sequence, two-period, steady-state study. Subjects received DTG at a dose of 50 mg (two 25 mg tablets) every 24 hours (q24h) for 5 days (period 1), and then the combination of morning DTG 50 mg and evening EFV 600 mg q24h was given for 14 days (period 2). There was no washout between treatment periods. All doses were administered in the fasting state. Serial PK plasma samples were collected after each period for DTG, and samples after period 2 were also collected for EFV. The metabolic profile of DTG was evaluated in plasma and urine using pooled samples for each subject [11] to assist in establishing the mechanism of any potential drug interaction (see Supplementary Material).

### Interaction with TPV/r

This was a phase I, open-label, single-sequence, three-period, steady-state study. Eighteen subjects received DTG 50 mg q24h for 5 days (period 1). Subjects were then administered a lead-inTPV/r 500/200 mg every 12 hours (q12h)for 7 days (period 2) followed by the combination of DTG 50 mg q24h and TPV/r 500/200 mg q12h for 5 days (period 3). There was no washout between treatment periods. All doses were administered with a moderate-fat meal. Serial PK samples for DTG were collected at the end of the dosing period of DTG alone (period 1) and DTG + TPV/r (period 3).

### Safety evaluations

In both studies, safety evaluations [adverse event (AE) reporting, clinical chemistry laboratory tests, urinalysis, vital signs and electrocardiogram] were performed throughout the study. Given the known hepatotoxicity profile of TPV/r, more frequent monitoring of liver chemistries was performed in the TPV/r study, and conservative stopping criteria were employed such that any subject reaching a grade 2 increase in alanine aminotransferase (ALT) or aspartate aminotransferase (AST) was discontinued from the trial. Both trials were conducted as inpatient studies with subjects housed in the unit for the duration of the study. The studies were conducted in accordance with the principles of the Declaration of Helsinki. A written informed consent was obtained from all subjects, and both protocols were approved by the institutional review board of the study sites.

### Bioanalytical methods

Dolutegravir and EFV were measured in plasma samples using validated methods, which consisted of extraction from plasma by protein precipitation with acetonitrile containing [^15^N^2^H_7_]dolutegravir or [^2^H_4_]-efavirenz as the internal standards, respectively. The plasma extracts were injected onto a 2.1 × 50 mm 3.5-micron XBridge™ C18 column (Waters Associates, Milford, MA, USA). Dolutegravir was eluted with a mobile phase consisting of 40 % acetonitrile in aqueous 0.1 % formic acid, and EFV was eluted with a mobile phase consisting of acetonitrile/0.1 % formic acid (65 % *v*/*v*) in a 5-mM ammonium acetate buffer containing 0.1 % acetic acid (35 % *v*/*v*). The eluates were detected by using a Sciex API 4000™ (AB Sciex, Framingham, MA, USA) equipped with a TurboIonSpray® ionisation source with multiple reaction monitoring (DTG positive ion mode *m*/*z* 420 > 277; internal standard *m*/*z* 428 > 277; EFV negative ion mode *m*/*z* 314 > 243; internal standard *m*/*z* 318 > 247). Data acquisition and processing were performed with Analyst 1.4.2 software (AB Sciex), and linear regression analysis calculations were performed using the Study Management System, SMS™ 2000 v.2.2 (GlaxoSmithKline, Research Triangle Park, NC, USA). The calibration range for DTG was 0.020 to 20 μg/mL and for EFV was 0.10 to 20 μg/mL. Quality control samples prepared separately at three concentrations were stored with study samples and analysed with each batch of samples against separately prepared calibration standards. The bias for the analysis of DTG was 4.0 to 8.2 % with precision values of 0.9 to 4.7 % (within-day) and ≤4.5 % (between-day). The bias for the analysis of EFV was 1.5 to 12.4 % with precision values of 0.6 to 5.3 % (within-day) and ≤4.4 % (between-day).

### Pharmacokinetic analysis

A non-compartmental PK analysis of the concentration-time data was performed with WinNonlin (version 5.2, Pharsight Corporation, Mountain View, CA, USA). Plasma PK parameters for both DTG and EFV were calculated using actual recorded times for each treatment. Parameters that were determined included AUC from time zero to the end of the dosage interval (AUC_(0–*τ*)_), maximum observed plasma concentration (*C*_max_), the time of maximum observed plasma concentration (*T*_max_) and plasma concentration at the end of the dosing interval (*C*_*τ*_).

### Statistical analysis

Statistical analyses were performed on the log-transformed PK parameters: AUC_(0–*τ*)_, *C*_*τ*_ and *C*_max_. Analysis of variance (ANOVA) was performed using SAS Mixed Linear Models procedure to assess the effect of EFV and TPV/r on the PK of DTG. Efavirenz PK data were compared with historical data. Subject was fitted as a random effect and treatment was fitted as a fixed effect in the model. The ratio of geometric least-squares means and associated 90 % confidence intervals were estimated for the PK parameters of interest. Dolutegravir given alone was considered as the reference treatment, and DTG co-administered with EFV or TPV/r was considered as the test treatment.

### Pharmacogenetic analysis

A blood sample was taken from 9 of 12 subjects in the EFV study who gave consent for pharmacogenetic analysis, and genomic DNA was extracted. Twenty-three polymorphisms from five genes/gene regions previously implicated in EFV metabolism were genotyped (Table 1). Only polymorphisms possibly conferring reduced enzyme activity were investigated. Due to the small number of samples available, no statistical modelling was planned or performed. However, the probabilities for each possible experimental outcome, based on the distribution of polymorphism carrier status between cases and controls and an assumption of no genetic association, were calculated a priori.

**Table 1:** Genetic polymorphisms evaluated

Gene	Polymorphisms
*CYP1A2*	*1C, *1K ,*7
*CYP2A6*	*2, *4, *5, *6, *7, *9, *11, *17, *20, *21
*CYP2B6*	*6, *8, *11, *16, *26, *27, *28
*CYP3A4/3A5*	*1B/*3, *6
*UGT2B7*	*2

## Results

### Demographics

#### Interaction with EFV

Twelve subjects were enrolled in the study and completed all treatment periods. One subject was prematurely discontinued from the study after period 2 due to non-compliance with the scheduled appointments. The median age for subjects was 36.5 years (range, 20–65 years), and all were male. Eleven of 12 subjects had White/Caucasian/European heritage, and the remaining subject was African American.

#### Interaction with TPV/r

Eighteen subjects were enrolled in the study. Five subjects withdrew early: four subjects due to an AE and another subject lost to follow-up. Fourteen subjects were male; the median age was 27.5 years (range, 19–45 years). Most subjects were either of White/Caucasian/European heritage (44 %) or African American heritage (39 %).

### Safety

#### Interaction with EFV

Repeat doses of DTG administered either alone or in combination with EFV were generally well tolerated. No deaths or serious AEs were reported, and no subject withdrew from the study due to an AE. Few AEs were reported, and all were mild (grade 1) to moderate (grade 2; *n* = 1) in intensity. Two of 12 subjects (17 %) reported at least one AE during DTG 50-mg once-daily dosing. Eleven of 12 subjects (92 %) reported at least one AE during DTG + EFV dosing; the most frequently reported AEs were dizziness [11 subjects (92 %)] and abnormal dreams [5 subjects (42 %)], which were considered by the investigator to be related to EFV based on its known psychiatric side effect profile. No clinically significant trends in laboratory abnormalities, vital signs or electrocardiogram values were evident. No grade 4 or drug-related grade 3 laboratory abnormalities were reported.

#### Interaction with TPV/r

The most frequently reported non-laboratory AEs were nausea [four subjects (22 %)] and vomiting [two subjects (11 %)] in the TPV/r group, followed by headache in the DTG group [two subjects (11 %)]. Four subjects had grade 2 increases in ALT, which resulted in premature discontinuation of the study based on protocol-defined stopping criteria. Three of these subjects also had grade 2 increases in AST. The AEs occurred in subjects receiving either TPV/r 500/200 mg or DTG 50 mg + TPV/r 500/200 mg. In all subjects, increases in ALT and AST began during dosing of TPV/r alone (period 2) and resolved with discontinuation of study medications.

### Pharmacokinetics

#### Interaction with EFV

**Fig. 1:**
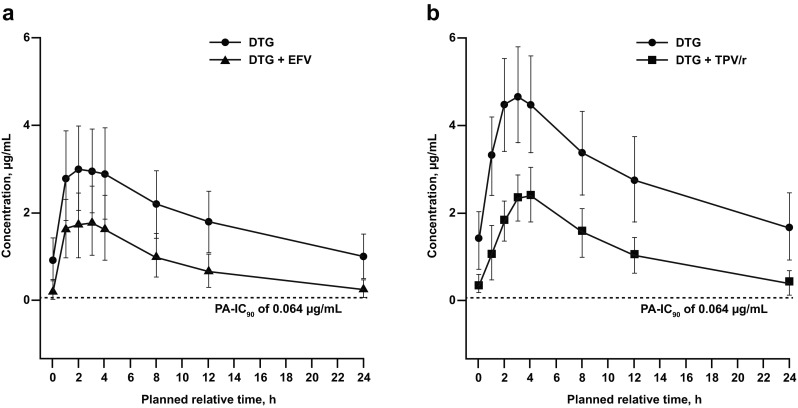
Mean concentration-time profiles of DTG with and without concomitant **a** EFV or **b** TPV/r. *DTG*, dolutegravir; *EFV*, efavirenz; *PA-IC*_*90*_, protein-adjusted 90 % inhibitory concentration; *TPV/r*, tipranavir/ritonavir

Pharmacokinetic parameters following repeat dose administration of DTG with and without EFV are shown in Table 2, and the mean concentration-time profiles are shown in Fig. 1. Co-administration of EFV 600 mg q24h and DTG 50 mg q24h resulted in 57, 39 and 75 % decreases in plasma DTG AUC_(0–*τ*)_, *C*_max_ and *C*_*τ*_, respectively. Plasma metabolic profiles of DTG were similar between the two treatment groups. Dolutegravir was the major component (>97 % of drug-related material) in the individual 24-h proportionally pooled plasma, while the ether glucuronide conjugate, M2, and a product of oxidation with sulfate conjugation, M11, were each present at mean values of <2 % of DTG in both treatment groups. A summary of the DTG-related components identified in urine and their relative ratio to total drug-related components are presented in Supplementary Table S1.

**Table 2:** Pharmacokinetic parameters of DTG with and without EFV or TPV/r and statistical analyses

Treatment	No.	*C*_max_ (μg/mL)	*T*_max_^a^ (h)	AUC_(0–*τ*)_ (μg · h/mL)	*C*_*τ*_ (μg/mL)
DTG 50 mg q24h	12	3.08 (30)	2.00 (1.0–4.0)	42.3 (39)	0.91 (53)
DTG 50 mg + EFV q24h	12	1.87 (42)	1.00 (1.0–4.0)	18.2 (50)	0.22 (76)
GLS mean ratio (90 % CI) DTG+EFV vs DTG alone	12	0.608 (0.506, 0.730)	ND	0.431 (0.346, 0.536)	0.245 (0.179, 0.336)
DTG 50 mg q24h	14	4.53 (23)	3.00 (2.00–3.00)	64.5 (28)	1.48 (40)
DTG 50 mg q24h + TPV/r 500/200 mg q12h	14	2.42 (23)	4.00 (1.00–4.00)	26.4 (30)	0.35 (54)
GLS mean ratio (90 % CI) DTG+TPV/r vs DTG alone	14	0.535 (0.500, 0.572)	ND	0.409 (0.379, 0.443)	0.239 (0.212, 0.270)

Pharmacokinetic parameters in geometric mean (CV%), except as noted

*AUC*_*(0–τ)*_ area under the concentration-time profile from time zero to the end of the dosage interval, *CI* confidence interval, *C*_*max*_ maximum observed plasma concentration, *C*_*τ*_ plasma concentration at the end of the dosing interval, *CV* coefficient of variation, *DTG* dolutegravir, *EFV* efavirenz, *GLS* geometric least-squares, *ND* not determined, *q24h* every 24 h, *T*_*max*_ time of maximum observed plasma concentration, *TPV/r* tipranavir/ritonavir

^a^Median (range)

#### Pharmacogenetics

Of the nine subjects who consented to pharmacogenetics research in the DTG/EFV interaction study, three subjects were classified as having high EFV exposure (cases), and six subjects had normal EFV exposure (controls). None of the polymorphisms evaluated in *CYP1A2*, *CYP2A6*, *CYP3A4/5* and *UGT2B7* accounted for the EFV elevation observed in the cases. However, there was evidence supporting a role of *CYP2B6* in EFV exposure in that all three cases were homozygous for the reduced function CYP2B6*6 polymorphism (Supplementary Figure S1), and this was an outcome that was predicted a priori to occur with a probability of 0.048 (results not shown). Individual plasma EFV PK parameters following repeat dose administration of EFV are summarised overall and by *CYP2B6* variant allele in Table 3. Subjects who carried CYP2B6*1/*1 or *1/*6 genotypes had EFV PK exposures similar to those in the EFV product label, which shows a mean AUC_(0–*τ*)_ of 184 μM·h (58.1 μg·h/mL), mean *C*_max_ of 12.9 μM (4.07 μg/mL) and mean minimum observed plasma concentration (*C*_min_) of 5.6 μM (1.77 μg/mL) [12]. Subjects who were CYP2B6*6 homozygous had two- to fourfold higher EFV exposures than those carrying CYP2B6*1/*1 or *1/*6 polymorphisms (Table 3).

**Table 3:** Pharmacokinetic parameters of EFV when co-administered with DTG and stratified by *CYP2B6* genotype

Genotype	No.	*C*_max_ (μg/mL)	*T*_max_^a^ (h)	AUC_(0–*τ*)_ (μg · h/mL)	*C*_*τ*_ (μg/mL)	*t*½ (h)
DTG 50 mg + EFV q24h						
All	12	6.02 (40)	2.50 (2.0–5.0)	84.2 (62)	2.41 (80)	22.2 (54)
CYP2B6*6 /*6^b^	3	9.50 (27)	2.00 (2.0–3.0)	174 (26)	5.85 (25)	39.1 (34)
CYP2B6*1/*1 or *1/*6 ^b^	6	4.52 (17)	2.5 (2.0–5.0)	55.7 (28)	1.45 (40)	15.6 (24)

Pharmacokinetic parameters in geometric mean (CV%), except as noted

*AUC*_*(0–τ)*_ area under the concentration-time profile from time zero to the end of the dosage interval, *C*_*max*_ maximum observed plasma concentration, *C*_*τ*_ plasma concentration at the end of the dosing interval, *CV* coefficient of variation, *DTG* dolutegravir, *EFV* efavirenz, *q24h* every 24 h, *t½* half-life;,*T*_*max*_ time of maximum observed plasma concentration

^a^Median (range)

^b^Nine of 12 study subjects provided a DNA sample and consent for pharmacogenetic research

#### Interaction with TPV/r

Pharmacokinetic parameters following repeat dose administration of DTG with and without TPV/r are shown in Table 2, and the mean concentration-time profiles are shown in Fig. 1. Co-administration with TPV/r resulted in 59, 46 and 76 % decreases in plasma DTG AUC_(0–*τ*)_, *C*_max_ and *C*_*τ*_, respectively (Table 2). 

## Conclusions

The drug interaction profile of DTG is characterised by few drug interactions requiring a dose adjustment. Dolutegravir does not induce or inhibit CYP isozymes and therefore does not cause interactions with drugs metabolised through these pathways [10]. As a victim of drug interactions, no clinically significant effects on DTG exposure have been observed with inhibitors of CYP3A or P-gp [7]. However, potent enzyme inducers such as rifampin and etravirine have been shown to significantly reduce DTG concentrations [9, 13]. Therefore, additional drug interaction studies with other antiretrovirals that may induce drug-metabolising enzymes were warranted.

Efavirenz is commonly used as a first-line agent in HIV treatment-naive subjects in combination with two nucleoside reverse transcriptase inhibitors. As such, it will not likely be a common partner with DTG in an antiretroviral regimen but may be co-administered in certain situations. Efavirenz has been well described to induce drug-metabolising enzymes and decrease plasma concentrations of a number of antiretrovirals and supportive medications [14].

The results of this study showed that co-administration with EFV resulted in 57, 39 and 75 % decreases in plasma DTG AUC_(0–*τ*)_, *C*_max_ and *C*_*τ*_, respectively. However, the magnitude of the change in DTG systemic exposure did not result in a change in the circulating metabolic profile. The profile after repeat administration to steady state was similar to that reported following a single dose [6]. The decrease in plasma DTG exposure is likely in part due to the induction of CYP3A4 and UGT. Induction of UGT is supported by the observation of an increase in glucuronide metabolites in the urine following EFV therapy (Supplementary Table S1).

Four of 12 subjects in the DTG/EFV interaction study showed higher EFV exposure than in historical data [12], and this was explained by carriage of the CYP2B6*6 polymorphism. Of these four subjects with high EFV exposures, three had DNA available for pharmacogenetic testing, and all were homozygous for CYP2B6*6. This polymorphism is known to result in a reduced function “poor metaboliser” status and is associated with high EFV concentrations [15]. Thus, it can be assumed that the unexpected higher EFV concentrations in these subjects observed in period 3 of this study (when DTG was co-administered with EFV) were due to carriage of CYP2B6*6 and not an effect of DTG on EFV PK. Such a finding is consistent with the knowledge that DTG should not affect the PK of CYP2B6 or CYP3A4 substrates. These four subjects had a mean DTG AUC that was similar to the group as a whole, further indicating no relationship between EFV and DTG exposure.

The construction of a new antiretroviral regimen with DTG for raltegravir (RAL)-resistant subjects may also require less commonly used agents such as TPV/r. In vivo, after a single dose, TPV/r moderately inhibits CYP3A4/5 and intestinal P-gp; after repeated dosing, TPV/r induces CYP3A4/5, UGT and P-gp [16, 17]. When TPV and ritonavir are combined, there is an approximate 40 % decrease in plasma ritonavir exposure, and thus the ritonavir dose is higher with TPV (200 mg) than with other protease inhibitors (100 mg) [18]. Due to its induction of drug-metabolising enzymes, concomitant use of TPV/r has been shown to decrease the Cmin of lopinavir, saquinavir and amprenavir by 52, 80 and 56 %, respectively [17].

Tipranavir/ritonavir decreased DTG exposure to a similar extent as did EFV, with decreases of 59, 46 and 76 % in DTG AUC_(0–*τ*)_, *C*_max_ and *C*_*τ*_, respectively. It has been shown to affect RAL as well, but to a lesser extent. Tipranavir/ritonavir decreased RAL AUC_(0–12)_, *C*_max_ and *C*_min_ by 24, 18 and 55 %, respectively [19]. Elvitegravir (EVG), which requires concomitant ritonavir administration, has also been studied with TPV in healthy subjects. Elvitegravir was administered as 200 mg once daily in combination with TPV/r 500/200 mg twice daily. No significant changes in EVG PK were observed [20]. Elvitegravir is primarily metabolised by CYP3A4, and the concomitant ritonavir may have overcome the enzyme-inducing effects of TPV. It should be noted that there are differences in DTG exposures between the studies (Table 2). This is because DTG was administered with food in the TPV study and administered fasting in the EFV study. As such, exposures in the TPV study were higher as food has been shown to increase the AUC of DTG by 33 to 66 % depending on the fat content [21].

The observed 75 % reduction in *C*_*τ*_ of DTG with EFV and TPV/r may be clinically significant. Even though doses as low as 10 mg once daily were effective in dose-ranging trials of DTG [22], the resulting exposures combined with PK variability may lead to a reduced response in some subjects. The clinical significance of the effects of EFV and TPV/r was evaluated in the phase III study SAILING [4], although data were limited. Subjects who received DTG 50 mg once daily in combination with EFV- or TPV/r-containing background regimens (*n* = 16) showed 78 % lower average DTG pre-dose (*C*_0_) concentrations (geometric mean of 0.186 μg/mL) and a lower week 48 Snapshot response rate (56.3 %) than subjects not on these inducers or inhibitors (atazanavir and atazanavir/ritonavir), who showed a response rate of 74.4 % [23]. Therefore, a higher DTG dose is needed when co-administration with EFV or TPV/r is required. Although a DTG dose higher than 50 mg once daily (e.g. 100 mg once daily or 50 mg twice daily) was not evaluated in the phase I studies reported here, PK data from the phase III study VIKING-3 [5] evaluating the DTG 50-mg twice-daily dose showed that DTG C0 (equivalent to *C*_τ_) in subjects receiving EFV and TPV/r in their background regimens (*n* = 18) had a geometric mean (CV%) of 1.62 μg/mL (63 %), which was 35 % higher than that observed in subjects receiving DTG 50 mg once daily without inducers in phase III studies in INI-naive populations (approximately 1.2 μg/mL) [23]. It is expected that DTG 50 mg twice daily with EFV or TPV/r will demonstrate a similar, if not higher, virologic response as that observed in subjects receiving DTG 50 mg once daily without these inducers. The safety profile for the DTG 50-mg twice-daily dose has been well established in VIKING-3 [5]. The DTG 100-mg once-daily dose is not recommended because of a less-than-dose-proportional increase in DTG exposure from the 50-mg dose to the 100-mg dose using the tablet formulation [24]. On the basis of these data, the dose of DTG in subjects receiving concomitant EFV or TPV/r in INI-naive subjects should be increased from 50 mg once daily to 50 mg twice daily. This recommendation is in line with that for another strong inducer, rifampin. Rifampin reduced DTG exposure to an extent similar to EFV, and TPV/r and DTG dose adjustment to 50 mg twice daily is suggested based on data showing that DTG *C*_τ_ from DTG 50 mg twice daily with rifampin is 22 % higher than that from DTG 50 mg once daily alone [13]. The use of EFV and TPV/r with DTG is expected to be low. It is unlikely for EFV to be given in subjects who are highly treatment resistant as it is widely considered as a “first-line” agent for treatment-naive subjects; however, there may be situations for its use in later stages of therapy. Tipranavir/ritonavir is more likely to be used in highly experienced subjects but only after better-tolerated options have been exhausted.

In INI-resistant subjects, the combination of DTG and EFV or TPV/r should be used with caution and based on resistance testing. When possible, alternative DTG-based regimens that do not include enzyme inducers should be considered in subjects with resistance to RAL and EVG.

## Electronic supplementary material

Below is the link to the electronic supplementary material.

ESM 1 (DOCX 19 kb)

ESM 2 (DOCX 15 kb)

ESM 3 (DOCX 161 kb)
