# Supplementary material for: Effects of enzyme inducers efavirenz and tipranavir/ritonavir on the pharmacokinetics of the HIV integrase inhibitor dolutegravir
Source: Eur J Clin Pharmacol. 2014 Aug 23;70(10):1173–9. doi: 10.1007/s00228-014-1732-8 (PMC4158172; doi:10.1007/s00228-014-1732-8)
Supplement: Supplementary file 1 — (DOCX 19 kb) [file 228_2014_1732_MOESM1_ESM.docx]

**Supplementary Material**

**Interaction with EFV**

**METHODS**

The metabolic profile of DTG was evaluated in plasma and urine to assist in establishing the mechanism of any potential drug interaction. Urine samples were collected at intervals of 0 to 12 hours and 12 to 24 hours after each treatment period. Individual urine pools were produced for each subject in a given treatment group by combining proportional volumes (% of total volume) from the 0- to 12-hours and 12- to 24-hours collections. Individual pooled human plasma samples were prepared for each subject and treatment group. Portions of plasma taken at each time point were combined by using volumes in proportion to the time interval between samples to create pools of human plasma representative of the area under the concentration-time profile (AUC) for each subject over the range 0 to 24 hours [1].

**Metabolic profiling of DTG**

Each pooled plasma sample was mixed with aqueous EDTA disodium salt solution (10 mg/mL) and subsequently extracted 3 times with methanol and acetonitrile. Pooled urine samples were lyophilised and the residue was reconstituted in water:methanol (90:10 v/v). Drug-related components in plasma extracts and urine samples were separated on a 100 × 4.6 mm 3.5-micron Symmetry C18 column (Waters Associates) at 40°C by a step-gradient elution method with a mobile phase composition of water:acetonitrile containing 0.1% ammonium acetate starting at 95:5 (v/v) and ending at 5:95 (v/v) over 50 minutes. The eluate was analysed by multistage mass spectrometry (MSn) using an LTQ Orbitrap^™^ Hybrid Mass Spectrometer with Xcalibur 2.07 software (Thermo Fisher Scientific, Bremen, Germany). Data-dependent scanning using an accurate mass list, consisting of all previously characterised metabolites, was used in addition to analysis of pre-dose samples to aid in the distinction of drug-related material from endogenous components.

**Mass spectral data analysis**

All metabolite estimations were established using mass spectral response. The mass spectral responses for each metabolite are reported as a percentage of the DTG response and as a percentage of the total mass spectral response for all drug-related components. This latter percentage represents the portion of each component as it is related to the total portion of all components. The comparison among components in each sample and between samples assumes all metabolites ionise equally. Although this premise is not strictly true, the noted trends (increases or decreases) observed for an individual component across subjects or between treatments should be valid. The mass spectral response factors from DTG and DTG glucuronide reference standards were used in the assessment.

**RESULTS**

**Urinary metabolic profile**

A summary of the DTG-related components identified in urine and their relative ratio to total drug-related components are presented in Supplementary Table S1. DTG and 11 metabolites were identified in individually pooled human urine. The metabolites identified in plasma and urine were structurally verified by high-resolution mass spectrometry from those previously characterised in humans [2] and from non-clinical studies. The urinary metabolites identified were a glucuronide conjugate (M2), the predominant drug-related component; a metabolite of benzylic oxidation (M3) and its hydrolytic N-dealkylation product (M1); oxidative defluorination with cysteine addition (M4); glucose conjugation (M9); pentose conjugation (M12); benzylic oxidation and glucuronidation (M8); fluorine substitution, sulfation (M10); oxidation, sulfation (M11); oxidative defluorination with cysteine addition and oxidation (M14); and glucuronidation with loss of carbon monoxide (M16). Quantitative differences in the relative amounts of M1 and M2 were observed between subjects administered DTG alone and DTG in combination with EFV. The mean M1 concentrations decreased, whereas the mean M2 concentrations increased following EFV treatment as compared with DTG administered alone. The other urinary metabolites did not display marked differences between the treatments.

**DISCUSSION**

The decrease in plasma DTG exposure is likely in part due to the induction of CYP3A4 and UGT. Induction of UGT is supported by the observation of an increase in glucuronide metabolites (particularly M2 and M8) in the urine following EFV therapy (Supplementary Table S1). Of the identified oxidative products, only M10, M11 and M4 appeared to increase with EFV administration, but each represented a small amount in the urine. Some of the low-level metabolites identified were not reported in the single-dose human mass balance study [2], but this could be due to mass spectral detection being more sensitive than radiochemical detection, the use of a higher dose, and dosing to steady state in this study.

**References**

1. Hop CE, Wang Z, Chen Q, Kei G (1998). Plasma**-**pooling methods to increase throughput for in vivo pharmacokinetic screening. J Pharm Sci 87:901–903
2. Castellino S, Moss L, Wagner D, et al (2013). Metabolism, excretion, and mass balance of the HIV-1 integrase inhibitor, dolutegravir, in humans. Antimicrob Agents Chemother 57:3536–3546

**Supplementary material for:** Effects of enzyme inducers efavirenz and tipranavir/ritonavir on the pharmacokinetics of the HIV integrase inhibitor dolutegravir

*European Journal of Clinical Pharmacology*

Ivy Song, Julie Borland, Shuguang Chen, Phyllis Guta, Yu Lou, David Wilfret, Toshihiro Wajima, Paul Savina, Amanda Peppercorn, Stephen Castellino, David Wagner, Louise Hosking, Michael Mosteller, Justin P. Rubio, Stephen C. Piscitelli

**Corresponding author:** Ivy H Song, GlaxoSmithKline; ivy.h.song@gsk.com
