# Supplementary material for: Effects of enzyme inducers efavirenz and tipranavir/ritonavir on the pharmacokinetics of the HIV integrase inhibitor dolutegravir
Source: Eur J Clin Pharmacol. 2014 Aug 23;70(10):1173–9. doi: 10.1007/s00228-014-1732-8 (PMC4158172; doi:10.1007/s00228-014-1732-8)
Supplement: Supplementary file 2 — (DOCX 15 kb) [file 228_2014_1732_MOESM2_ESM.docx]

**Supplementary Table S1.** Summary of the percentage of DTG and its metabolites in

pooled urine relative to DTG and total identified drug-related response

| **DTG/ Metabolite** | **% DTG** | | **% Total drug-related response** | |
| --- | --- | --- | --- | --- |
|  | **DTG 50 mg q24h** | **DTG 50 mg + EFV q24h** | **DTG 50 mg q24h** | **DTG 50 mg + EFV q24h** |
| DTG | 100 | 100 | 6.4 (1.8) | 6.2 (5.0) |
| M1 | 217 (186) | 66.1 (55.9) | 12.2 (8.6) | 3.1 (2.6) |
| M2 | 1,188 (275) | 1,633 (607) | 72.8 (10.9) | 79.8 (9.3) |
| M3 | 51.9 (57.3) | 38.8 (48.6) | 2.9 (2.6) | 1.9 (2.4) |
| M4 | 24.3 (18.7) | 47.2 (29.8) | 1.4 (0.8) | 2.3 (1.5) |
| M8 | 5.8 (5.5) | 14.3 (12.3) | 0.3 (0.2) | 0.8 (0.8) |
| M9 | 23.1 (9.2) | 20.7 (6.5) | 1.4 (0.5) | 1.4 (1.5) |
| M10 | 28.0 (62.2) | 39.8 (55.8) | 1.6 (3.6) | 2.1 (3.1) |
| M11 | 12.2 (8.9) | 41.3 (34.4) | 0.7 (0.4) | 2.0 (1.7) |
| M12 | 2.6 (1.8) | 1.0 (1.2) | 0.2 (0.1) | <0.1 |
| M14 | 1.2 (2.0) | 2.5 (2.8) | 0.1 (0.1) | 0.1 (0.1) |
| M16 | 2.3 (1.7) | 2.9 (2.2) | 0.1 (0.1) | 0.1 (0.1) |

DTG, dolutegravir; q24h, every 24 hours.

n=12. Values are mean (standard deviation).

**Supplementary material for:** Effects of enzyme inducers efavirenz and tipranavir/ritonavir on the pharmacokinetics of the HIV integrase inhibitor dolutegravir

*European Journal of Clinical Pharmacology*

Ivy Song, Julie Borland, Shuguang Chen, Phyllis Guta, Yu Lou, David Wilfret, Toshihiro Wajima, Paul Savina, Amanda Peppercorn, Stephen Castellino, David Wagner, Louise Hosking, Michael Mosteller, Justin P. Rubio, Stephen C. Piscitelli

**Corresponding author:** Ivy H Song, GlaxoSmithKline; ivy.h.song@gsk.com
