# Supplementary material for: Effects of enzyme inducers efavirenz and tipranavir/ritonavir on the pharmacokinetics of the HIV integrase inhibitor dolutegravir
Source: Eur J Clin Pharmacol. 2014 Aug 23;70(10):1173–9. doi: 10.1007/s00228-014-1732-8 (PMC4158172; doi:10.1007/s00228-014-1732-8)
Supplement: Supplementary file 3 — (DOCX 161 kb) [file 228_2014_1732_MOESM3_ESM.docx]

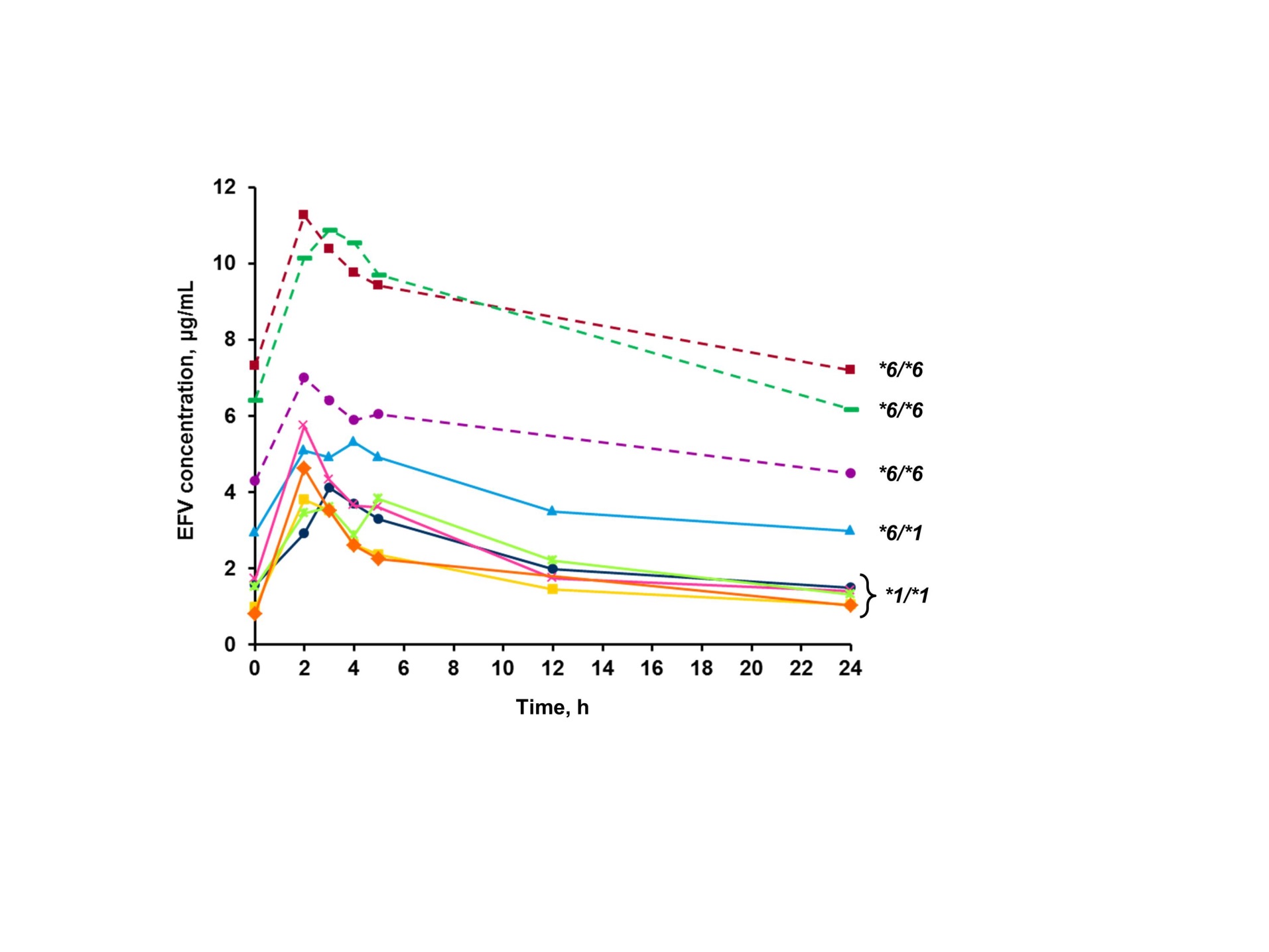


Supplementary Fig S1 EFV exposure over time by *CYP2B6*6* genotype status for patients that provided consent for pharmacogenetics research (n=9). Three of 9 subjects (all of whom were *CYP2B6*6* homozygous) had high EFV exposure, indicated by the dashed lines. EFV, efavirenz

**Supplementary material for:** Effects of enzyme inducers efavirenz and tipranavir/ritonavir on the pharmacokinetics of the HIV integrase inhibitor dolutegravir

*European Journal of Clinical Pharmacology*

Ivy Song, Julie Borland, Shuguang Chen, Phyllis Guta, Yu Lou, David Wilfret, Toshihiro Wajima, Paul Savina, Amanda Peppercorn, Stephen Castellino, David Wagner, Louise Hosking, Michael Mosteller, Justin P. Rubio, Stephen C. Piscitelli

**Corresponding author:** Ivy H Song, GlaxoSmithKline; [ivy.h.song@gsk.com](mailto:ivy.h.song@gsk.com)
